# Sustainable Development of Polish Macroregions—Study by Means of the Kernel Discriminant Coordinates Method

**DOI:** 10.3390/ijerph17197021

**Published:** 2020-09-25

**Authors:** Mirosław Krzyśko, Waldemar Wołyński, Waldemar Ratajczak, Anna Kierczyńska, Beata Wenerska

**Affiliations:** 1 Interfaculty Institute of Mathematics and Statistics, Calisia University-Kalisz, 62-800 Kalisz, Poland; mkrzysko@amu.edu.pl; 2 Faculty of Mathematics and Computer Science, Adam Mickiewicz University, 61-614 Poznań, Poland; 3 Faculty of Social Sciences, Calisia University-Kalisz, 62-800 Kalisz, Poland; walrat@amu.edu.pl (W.R.); b.wenerska@pwsz.kalisz.pl (B.W.); 4 Faculty of Economic, The Great Poland Socio-Economic University, 63-000 Środa Wlkp., Poland; a.kierczynska@onet.pl

**Keywords:** sustainable development, spatio-temporal data, NUTS-1, kernel discriminant coordinates method, super macroregions

## Abstract

The aim of this study was to investigate if the macroregions of Poland are homogeneous in terms of the observed spatio-temporal data characterizing their sustainable development. So far, works related to the sustainable development of selected territorial units have been based on data relating to a specific year rather than many years. The solution to the problem of macroregion homogeneity goes through two stages. In step one, the original spatio-temporal data space (matrix space) was transformed into a kernel discriminant coordinates space. The obtained kernel discriminant coordinates function as synthetic measures of the level of sustainable development of Polish macroregions. These measures contain complete information on the values of 27 diagnostic features examined over 15 years. In the second step, cluster analysis was used in order to identify groups of homogeneous macroregions in the space of kernel discriminant coordinates. The agglomeration method and the Ward method were chosen as commonly used methods. By means of both methods, three super macroregions composed of homogeneous macroregions were identified. Within the kernel discriminant coordinates, the differentiating power of a selected set of 27 features characterizing the sustainable development of macroregions was also assessed. To this end, five different and most commonly used methods of discriminant analysis were used to test the correctness of the classification. Depending on the method, the classification errors amounted to zero or were close to zero, which proves a well-chosen set of diagnostic features. Although the data relate only to a specific country (Poland), the presented statistical methodology is universal and can be applied to any territorial unit and spatial-temporal dynamic data.

## 1. Introduction

The concept of sustainable development played an important role in the second half of the 20th century. The concept was first defined in a report of the World Commission on Environment and Development, “Our Common Future”, under the supervision of G. Brundtland (Our Common Future, UN New York, 1987). Sustainable development is “development that meets the needs of today and does not limit the ability of future generations to meet their own needs.” According to the report, this means that “all future generations have the right to live and enjoy all the values of the environment known to them, just like you, or even better.” In other words, it is a socio-economic development in which the forms and dynamics of economic activity, institutions, lifestyles and the population size are such that the existing and future generations will be provided with an adequate standard of living, and all aspects of this development are secured by the availability of natural resources, ecosystems and life support systems. This means that the economic development of the current generation should not happen at the cost of the depletion of non-renewable stocks (Strategy for the sustainable development of Poland until 2025, Ministry of the Environment, Warsaw, 1999).

Sustainable development has been defined in a variety of ways (Awan et al. [1], Awan et al. [2], Borys [3]). In our opinion, it is based on the following four pillars: 1. Demography and social capital, 2. Production, services and trade, 3. Public finance, and 4. The environment and protection thereof. “Sustainability” has become a global buzzword as a potential solution to many international, regional, and local problems of contemporary society: over-population, diseases, political conflicts, infrastructure deterioration, pollution, and unlimited urban expansion accompanied by limited availability of resources. The United Nations World Commission on Environment and Development (WECD, 1987) coined a definition of sustainable development that is probably the most well-known in all of the literature on sustainability: “development that meets the needs of the present without compromising the ability of future generations to meet their own needs”.

Along with the concept of sustainable development, the related term sustainable regional development appeared as a traditional goal of the state’s regional policy. This applies to activities of public administration bodies that should aim at counteracting excessive or unjustified interregional differences.

Regional development is usually defined as integral community development (social, economic, environmental and healthcare, technological, cultural and recreational) in a particular territory. Regional development must be based on its optimal expansion constituents (social, natural and economic development aspects) aimed at maintaining a certain standard of living and improvement of its quality through the mentioned constituents. Regional development encompasses not only traditional policy in a specific territory, but also a socioeconomic process taking place in a specific political and cultural context (Atkinson [4], Bourdeau [5], Spangenberg [6], OECD [7], Wheeler and Beatley [8], Wong [9]). Regional development in today’s context is at a critical juncture, with multiple crises (financial, food and energy) forcing us to re-assess the economic paradigm of our time and to evaluate how to better address the unfulfilled promises that we are currently leaving to future generations in the areas of employment, social progress, quality of life and respect for nature. While there is no doubt about the importance of integrating the pillars of sustainable development on the regional level, implementation of this concept has proved challenging in practice. In fact, integration of the environmental, economic, and social dimensions of sustainable development on a regional level implies the implementation of complimentary and coordinated actions in different areas. This results in economic growth that is also supposed to achieve social objectives, without posing a threat to the rare resources of the planet. Effective integration of these four dimensions (pillars) requires implementation of a set of focused and specific actions that are complementary and fit into an overarching sustainable development framework.

Previous papers related to the sustainable development of regions were based on data for a specific year or, for comparison purposes, for two specific years. If the authors of these papers chose, say, *p* features describing sustainable development of regions, then the obtained data were *p*-dimensional vectors or points in p-dimensional Euclidean space. Then, from these vectors numerical indicators (linear combinations) were constructed, considered to be the characteristics of the sustainable development of regions (Kontostanou-Karalivanou et al. [10], United Nations [11], Streimikiene [12], Dočekalová et al. [13], Jovovic et al. [14], Roszkowska and Wyszkowska [15]). An extensive overview of sustainability performance evaluation, including a literature review and future directions, was presented by Büyüközkan and Karabulut [16], Krasławski et al. [17].

This paper deals with a more general issue and presents a proposal for the regionalization of spatio-temporal data, which are more general than vector data considered at a given moment. We also propose a new way of constructing numerical indicators describing the sustainable development of specific territorial units.

The paper is based on data concerning Poland. The country is administratively divided into seven macroregions composed of voivodships (16 voivodships in total), and each voivodeship is further divided into poviats. The term poviat, defining a given territorial unit, is used by Statistics Poland, which is associated with EUROSTAT. In total, there are 379 poviats in Poland. Each poviat has been described by means of 27 features, divided into four pillars that characterize the sustainable development of this territorial unit. These features were observed in the years 2002–2016, i.e., for 15 years. Our data can be written in the form of a matrix with 15 rows and 27 columns. This type of data is called spatio-temporal data. They are dynamic and more general than static vector data. The choice of Poland as the research area stemmed from the availability of data. The data concerns 379 poviats characterized by 27 features observed over 15 years. The data therefore consist of 153,495 numbers, a very large data set that is not easy to collect. The data were provided by the Local Data Bank of Statistics Poland. On the other hand, when selecting Poland as the research area, the authors of the study had in mind the usefulness of the obtained results for Polish governmental decision-makers.

The aim of this paper is to investigate if Polish macroregions are homogenous in terms of spatio-temporal data characterizing their sustainable development.

To solve this problem by means of statistical methodology, two multidimensional statistical methods were used: discriminant analysis and cluster analysis. The following solutions were adopted.

In the first step, the original spatio-temporal data space (matrix space) was transformed into a vector space of discriminant coordinates. This transformation takes place in two ways known in the discriminant analysis literature: classical discriminant coordinates (Fisher [18], Rao [19]) and kernel discriminant coordinates, as first described by Baudat and Anouar [20]. Both transformations were developed for fixed vector data. Since our data are a matrix, it was necessary to extend these transformations to the matrix case. This extension proved troublesome in the case of Fisher’s classical discriminant coordinates. Hence the choice of kernel discriminant coordinates, since in their case the said extension was possible. The space of kernel discriminant coordinates is a space of dimension min(p,c−1), where *p* is the number of observed features and *c* is the number of macroregions. Our case consists of a six-dimensional space, so the dimension of the space in which further inference will take place is relatively low.

We treated the obtained kernel discriminant coordinates further as synthetic measures of the level of sustainable development of Polish macroregions. These measures contain complete information on the values of 27 diagnostic features measured over 15 years. Each of the six kernel discriminant coordinates was given a different power to differentiate macroregions. In the space of the first two kernel discriminant coordinates, it is possible to graphically present the mutual position of the studied macroregions.

In the space of kernel discriminant coordinates, it is also easy to assess the differentiating power of a selected set of features characterizing individual poviats. To this end, various methods of discriminant analysis can be used, verifying the correctness of the classification of individual poviats into the seven distinguished macroregions. In this paper, five different, most commonly used methods of discriminant analysis were used. The percentage of misclassifications of individual poviats to seven macroregions was calculated using these methods. Zero or close to zero classification errors indicate a well-chosen set of diagnostic features.

In the second step, cluster analysis was used in order to select groups of homogeneous macroregions in the six-dimensional space of kernel discriminant coordinates. The agglomeration method and the Ward method were chosen as commonly used methods. As a result, three super macroregions composed of homogeneous macroregions were identified.

According to the authors, the proposed statistical methodology is the main value of this paper. Although the data relate only to a specific country, the presented statistical methodology is universal and can be applied to any territorial unit and spatio-temporal dynamic data.

The paper is organized as follows: Section 2 contains a presentation of spatial units and data. Section 3 describes the statistical methodology. Section 4 contains the research results. Concluding remarks are provided in Section 5.

## 2. Spatial Units and Data

Achieving the research goal adopted in this work required collecting data characterizing various aspects of sustainable development for N=379 poviats belonging to one of the c=7 macroregions. The macroregions are NUTS-1 units according to the EUROSTAT nomenclature (2018).

**Figure 1:**
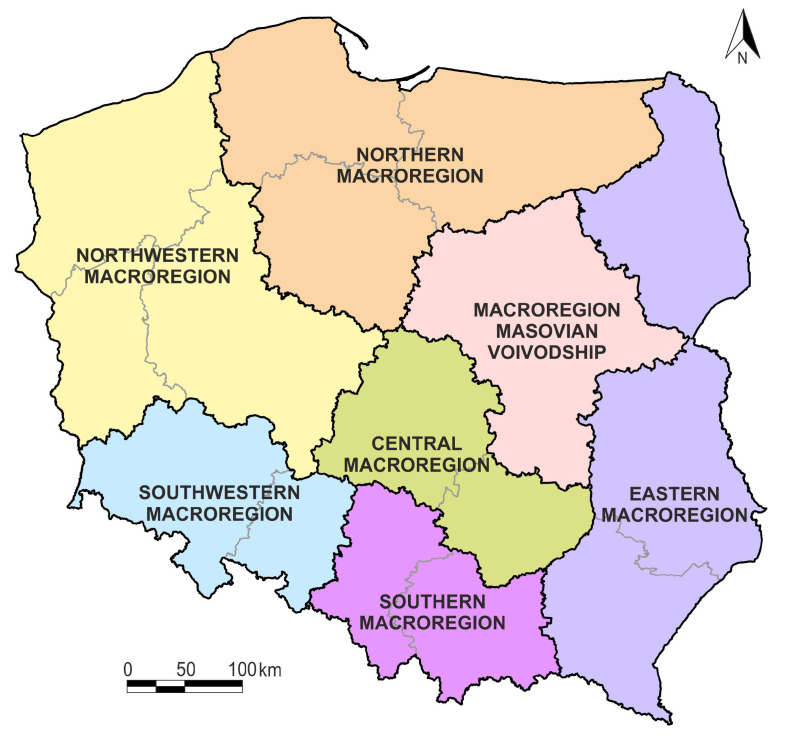
Macroregions in Poland (NUTS-1. 2018). Source: Statistics Poland.

The structure of NUTS-1 in Poland is presented in Figure 1 and Table 1.

A set of p=27 variables (local data bank information of Statistics Poland was used) was collected for each poviat, for the period of 2002–2016, i.e., T=15 years. They were divided into four pillars relating to various areas of sustainable development, i.e., 1. Social capital, 2. Production, services and trade, 3. Finance, 4. The environment and protection thereof (see Table 2). The choice of the variables was made on the basis of their merits and in accordance with the authors’ previous experience. The accuracy of the selection of variables was confirmed using discriminant analysis methods (see Section 4).

Therefore each poviat was characterized by an matrix of 15×27 size.

## 3. Statistical Methodology

### 3.1. Kernel Discriminant Coordinates for Spatio-Temporal Data

The main idea of the kernel-based methods is to map the input data to a feature space through nonlinear mapping where the inner products in the feature space can be computed by a kernel function while the nonlinear mapping is not known explicitly. Kernel Fisher discriminant analysis (KFD) provided by Baudat and Anouar [20] and the generalized discriminant analysis (GDA) provided by Mika et al. [21] are two independently developed approaches for kernel-based nonlinear extensions of discriminant coordinates. They are essentially equivalent. The method is described in a book by Shawe-Taylor and Cristianini [22]. To avoid confusion, we will refer to this approach as kernel discriminant coordinates analysis (KDCA). These new variables are also sometimes called kernel canonical variates yet the name is misleading because kernel canonical variables with completely different properties occur in the kernel canonical analysis. Another name is “kernel discriminant functions”, which is inappropriate because discriminant functions are surfaces that separate the *c* classes from one another.

In this paper, the authors present a proposal of extending vector kernel analysis to matrix kernel analysis in their original way. Obviously, in the present case the data are spatio-temporal. The kernel discriminant coordinates analysis is a method developed for fixed vector data. This paper includes an extension of this method to spatio-temporal data. In this case, each object is characterized by a (T×p)-size matrix X containing the *p* values observed at *T* moments.

Let $\left\{\left(x_{1},y_{1}\right),\ldots ,\left(x_{N},y_{N}\right)\right\}$, $x_{i}\in R^{T\times p}$, $y_{i}\in R^{c}$, i=1,…,N, be a data set.

The input space $R^{T\times p}$ is now mapped nonlinearly into a feature space $H_{k}$:

$$\phi :R^{T\times p}\to H_{k}$$

where ϕ is the mapping function that maps the input space to the reproducing kernel Hilbert space (RKHS) $H_{k}$. Please note that $H_{k}$ could have arbitrarily large, possibly infinite dimensionality. The vector $\phi \left(x_{i}\right)={\tilde{x}}_{i}$ is called the feature vector corresponding to the observation $x_{i}\in R^{T\times p}$, i=1,…,N.

The nonlinear transformation ϕ is in general unknown; however, we select a known form of the nonnegative definite kernel function:

$$k\left(x_{i},x_{j}\right)={\langle \phi \left(x_{i}\right),\phi \left(x_{j}\right)\rangle }_{H_{k}},\;i,j=1,2,\ldots ,N.$$

A kernel function can be interpreted as a kind of similarity measure between the matrices $x_{i}$ and $x_{j}$.

Throughout this paper we use the Gaussian kernel:

$$k\left(x_{i},x_{j}\right)=\exp(-\gamma ∥x_{i}-x_{j}{∥}_{F}^{2}),\;\gamma >0,$$

where

$${∥A∥}_{F}=\sqrt{\mathrm{tr}(A^{⊤}A)}$$

is the Frobenius norm. The constant γ>0 is appropriately selected from the data. We take into account the lower-triangular matrix, which has its (i,j)-th element given by $∥x_{i}-x_{j}{∥}_{F}^{2}$, i,j=1,…,N. The value of γ was used as the reciprocal of the arithmetic mean of the elements of this matrix.

Similarly, as in the classical case of DCA, in kernel discriminant analysis we find, for a *c*-class problem, the c−1 vectors onto which the projections of the data of one class are maximally separated from the remaining classes in the feature space.

Let $\tilde{m}=\frac{1}{n}\sum_{i=1}^{n}{\tilde{x}}_{i}$ be the sample mean, and let ${\tilde{m}}_{j}=\frac{1}{n_{j}}\sum_{i\in V_{j}}{\tilde{x}}_{i}$ be the mean of the *j*th class, for j=1,…,c, in the feature space.

By ${\tilde{S}}_{b}$ and ${\tilde{S}}_{t}$ we denote the between-class and total scatter matrices in the feature space, respectively.

We have:

$${\tilde{S}}_{b}=\sum_{j=1}^{c}n_{j}{\langle \mu_{j},\mu_{j}\rangle }_{H_{k}},\;{\tilde{S}}_{t}=\sum_{j=1}^{n}{\langle v_{j},v_{j}\rangle }_{H_{k}},$$

where

$$\mu_{j}={\tilde{m}}_{j}-\tilde{m},\;v_{j}={\tilde{x}}_{j}-\tilde{m_{j}},\;j=1,\ldots ,n.$$

KDCA seeks vectors $b_{i}$(i=1,…,c−1) that maximize the ratio of between-class scatter and total scatter for maximum class separation.

Namely, we want to maximize the objective function:

$$\begin{array}{c} J\left(b_{i}\right)=\frac{b_{i}^{⊤}{\tilde{S}}_{b}b_{i}}{b_{i}^{⊤}{\tilde{S}}_{t}b_{i}}, \end{array}$$

subject to an additional restriction:

$$\begin{array}{c} b_{i}^{⊤}{\tilde{S}}_{t}b_{j}=\delta_{ij},\;i,j=1,\ldots ,c-1. \end{array}$$

Finding the directional vectors $b_{i}$ reduces to solving the following generalized eigenvalue problem:

(1) $$\begin{array}{c} {\tilde{S}}_{b}b_{i}=\lambda_{i}{\tilde{S}}_{t}b_{i},\;b_{i}^{⊤}{\tilde{S}}_{t}b_{j}=\delta_{ij}. \end{array}$$

Now, we show how to solve problem (1) without knowing the explicit representation of the mapping ϕ and the feature space $H_{k}$ and without forming ${\tilde{S}}_{b}$ and ${\tilde{S}}_{t}$ explicitly.

Please note that any vector $b\in R^{N}$ can be represented as:

$$b=b_{1}+b_{2}$$

where $b_{1}\in \mathrm{span}\left\{{\tilde{x}}_{1},\ldots ,{\tilde{x}}_{N}\right\}$ and $b_{2}\in \mathrm{span}{\left\{{\tilde{x}}_{1},\ldots ,{\tilde{x}}_{N}\right\}}^{⊥}$, and ${\tilde{S}}_{b}b_{2}=0$ and ${\tilde{S}}_{t}b_{2}=0$ for any $b_{2}\in \mathrm{span}{\left\{{\tilde{x}}_{1},\ldots ,{\tilde{x}}_{N}\right\}}^{⊥}$.

Therefore, for any vector b satisfying (1),

$${\tilde{S}}_{b}b_{1}={\tilde{S}}_{b}\left(b_{1}+b_{2}\right)=\lambda {\tilde{S}}_{t}\left(b_{1}+b_{2}\right)=\lambda {\tilde{S}}_{t}b_{1}.$$

Hence we can restrict the solution space for (1) to $\mathrm{span}\{{\tilde{x}}_{1},\ldots ,{\tilde{x}}_{N}\}$.

Let b be represented as a linear combination of ${\tilde{x}}_{i}$, i=1,…,N, $b=\sum_{i=1}^{N}\alpha_{i}{\tilde{x}}_{i}$ and $\alpha ={\left(\alpha_{1},\ldots ,\alpha_{N}\right)}^{\prime }$.

Hence, the generalized eigenvalue problem

$${\tilde{S}}_{b}b=\lambda_{i}{\tilde{S}}_{t}b$$

is equivalent to

$$\begin{array}{c} \tilde{K}D\tilde{K}\alpha =\lambda \tilde{K}\tilde{K}\alpha , \end{array}$$

where

$$\tilde{K}=HKH,$$

$K=(k\left(x_{i},x_{j}\right))$, i,j=1,…,N, $H=I_{N}-\frac{1}{N}1_{N}1_{N}^{⊤}$, $1_{N}\in R^{N}$ and the matrix D is defined by

$$D_{ij}=\left\{\begin{array}{cc} \frac{1}{n_{k}}, & \mathrm{if}\;x_{k}\;\mathrm{and}\;x_{j}\;\mathrm{belong}\;\mathrm{to}\;\mathrm{the}\;k\mathrm{th}\;\mathrm{class},\\ 0, & \mathrm{otherwise}. \end{array}\right.$$

Suppose the rank of K is *r*(r≤N). The projection of observation x onto b in $H_{k}$ is given by:

$$\begin{array}{c} \langle b,\phi \left(x\right)\rangle =\sum_{i=1}^{N}\alpha_{i}\langle \phi \left(x_{i}\right),\phi \left(x\right)\rangle =\sum_{i=1}^{N}\alpha_{i}k\left(x_{i},x\right). \end{array}$$

Solving the generalized eigenvalue problem presents certain difficulties because both matrices $\tilde{K}D\tilde{K}$ and $\tilde{K}\tilde{K}$ are nonnegative definite. Krzyśko et al. [23] compared six algorithms that overcome these difficulties in different ways. The used performance quality criterion was the percentage of misclassification using the linear discriminant function in the spaces of kernel discriminant coordinates. The running time of the procedures was also recorded.

The mentioned authors recommended the algorithms proposed by Baudat and Anouar [20], Cai et al. [24] and two algorithms proposed by Zhang et al. [25]. They present similar classification errors and have comparable running times.

### 3.2. Cluster Analysis

Cluster analysis methods were applied to identify areas with similar degrees of sustainable development. Two methods were used, namely the Ward method and the agglomeration method (see, for example, Seber [26], Chapter 7, Mirkin [27], and Krzyśko et al. [28], Chapter 12).

The cluster procedure is based on the Mahalanobis distance between the macroregions to which individual poviats were satisfactorily qualified. This distance is defined by the following formula:

$$d_{ij}^{2}={\left({\bar{x}}_{i}-{\bar{x}}_{j}\right)}^{⊤}S^{-1}\left({\bar{x}}_{i}-{\bar{x}}_{j}\right),$$

where

$${\bar{x}}_{i}=\frac{1}{N_{i}}\sum_{j=1}^{N_{i}}x_{ij},\;i=1,2,\ldots ,c,$$

and

$$S=\frac{1}{N-c}\sum_{i=1}^{c}\sum_{j=1}^{N_{i}}\left(x_{ij}-{\bar{x}}_{i}\right){\left(x_{ij}-{\bar{x}}_{i}\right)}^{⊤},\;N=N_{1}+\ldots +N_{c}.$$

## 4. Research Results

In the first step, the original data written in the form of N=379 matrices (corresponding to the individual poviats) of the size of T×p, where p=27 is the number of features characterizing various aspects of sustainable development of poviats and T=15 is the number of subsequent years in which these features were observed (years 2002–2016), were transformed into c−1=6 kernel discriminant coordinates, where c=7 is the number of macroregions in question. The structure of kernel discriminant coordinates for spatio-temporal data is described in Section 3.1. We went from matrix space to ordinary Euclidean space. The space of kernel discriminant coordinates is a space of dimension min(p,c−1), where *p* is the number of observed features, and *c* is the number of macroregions. In our case, it is a six-dimensional space, so the dimension of the space in which further inference will take place is relatively small, as was previously mentioned.

We treat the obtained kernel discriminant coordinates further as indicators (synthetic measures) of the level of sustainable development of Polish macroregions. These indicators contain full information on the values the 27 diagnostic features measured over 15 years, which characterize the sustainable development of poviats and consequently the macroregions that include individual poviats. They are therefore composed indicators of sustainable development.

**Figure 2:**
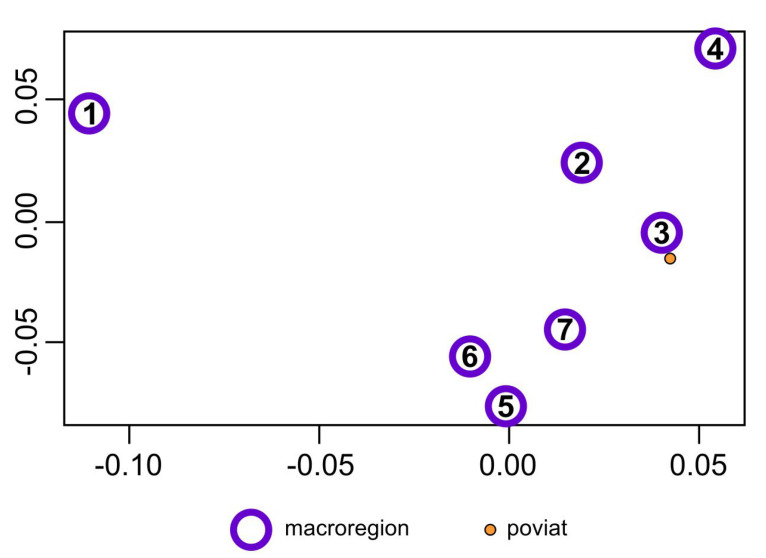
Macroregions in the first and second KDC systems and poviats recognized as belonging to specific macroregions. Source: own compilation.

These six synthetic indicators have a different power of differentiating the studied macroregions (these indicators have different variances). The first indicator is the strongest and the sixth indicator is the least powerful. It is not possible to see the mutual position of the most important macroregion in the six-dimensional space of these indicators, but it is possible in the space of the first two composed indicators that differentiate the macroregions most potently. Figure 2 shows the mutual position of the seven macroregions in the system of the first two composed indicators (kernel discriminant coordinates).

In the full six-dimensional space of kernel discriminant coordinates, it is also easy to assess the differentiating power of a selected set of 27 features characterizing individual poviats. To this end, various methods of discriminant analysis can be used, examining the correctness of the classification of individual poviats into the seven identified macroregions.

In this paper, five different, most commonly used, methods of discriminant analysis were used (see, for example, Wasserman [29], Chapter 22, Krzyśko et al. [28], Chapters 1 and 4–6, Hastie et al. [30]): linear discriminant function (LDF), naive Bayes normal classifier (NB (Normal)), K-nearest neighbors method (KNN), classification trees (CART) (Tree (CART)) and support vector machines (SVM). Table 3 presents the percentage of misclassifications of individual poviats into seven macroregions, using these methods, and the estimated ten-fold cross validation method (10-cv). Clearly, zero or close to zero classification errors indicate a well-chosen set of 27 diagnostic features. This result was corroborated by our substantive selection of features characterizing sustainable development of macroregions with statistical arguments.

In the second step, cluster analysis was used in order to select groups of homogeneous macroregions in the six-dimensional space of kernel discriminant coordinates. The agglomeration method and the Ward method were selected as commonly used methods. The Mahalanobis distance was chosen as a measure of the distance between the mean vectors of individual macroregions. It takes into account not only the difference between the mean vectors of two macroregions; the difference is also weighted by the variances and covariances of the examined features estimated for a total of seven macroregions (the differentiation of poviats around the mean macroregions was taken into account). The Mahalanobis distances between macroregions are shown in Table 4.

**Figure 3:**
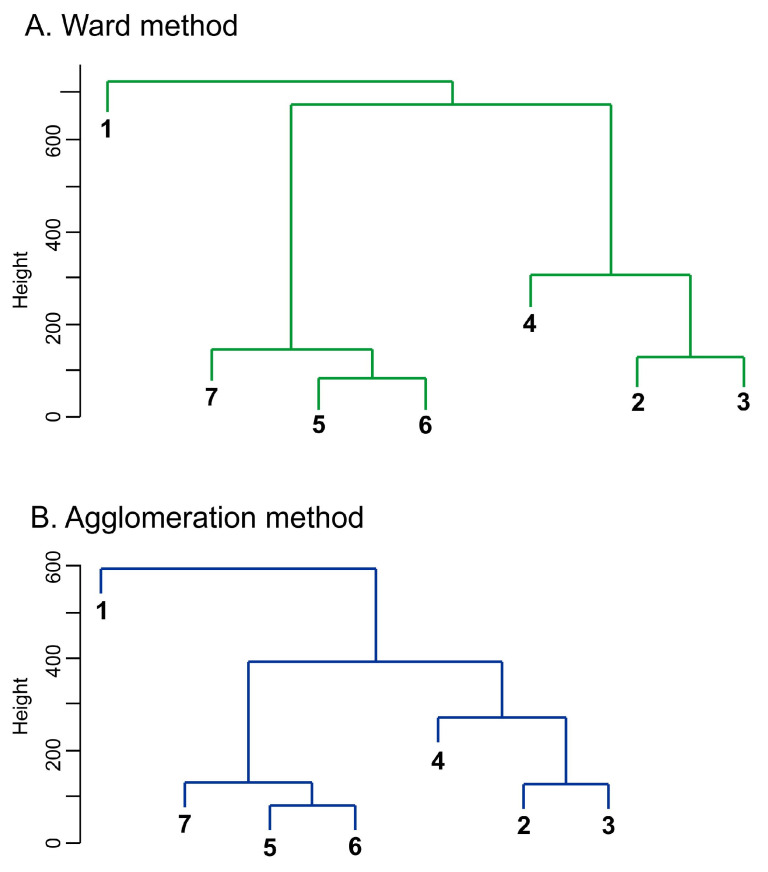
Grouping of macroregions with cluster methods (1—southern, 2—northwestern, 3—southwestern, 4—northern, 5—central, 6—eastern, 7—Masovian voivodship). Source: own compilation.

On the basis of the Mahalanobis distances, dendrograms were constructed using the agglomeration method and the Ward method to identify areas with similar degrees of sustainable development. Both provided results identical to the ones illustrated in Figure 3.

Three areas were found with a high degree of internal similarity considered for sustainability. Two of them can be referred to as super macroregions.

Super macroregion (area) I consist of the following macroregions:

2—northwestern,

3—southwestern,

4—northern.

Super macroregion (area) II:

5—central,

6—eastern,

7—Masovian voivodship.

The third area represents a separate southern macroregion (I).

The delimitated super macroregions vary with respect to area, territorial capital and socio-economic development specified by the values of the Human Development Index (HDI).

Super macroregion I consists of three macroregions, eight regions (voivodships) and 175 sub-regions (poviats). It represents 50.1% of Poland’s total area.

Its three regions, i.e., Great Poland, Lower Silesia and Pomerania, belong to a set of five regions of Poland that enjoy the highest GDP in the long run. By contrast, the Poznań poviat is the most developed poviat in Poland in socio-economic terms (Kierczyńska [31]).

Notably, super macroregion I includes regions with a lower degree of economic development but with enormous natural environment resources. These regions include Warmian-Masurian, Pomeranian, West-Pomeranian and Lubusz. This diversity within the super macroregion translates into the average HDI value of super macroregion I, determined for 2015, amounting to 0.864. It is lower than the average HDI value of super macroregion II and the southern macroregion (Hozer-Koćmiel [32]).

Super macroregion II also consists of three macroregions, including six voivodships and 146 poviats. It covers 41.1% of Central and Eastern Poland. Its seventh macroregion (Masovian voivodship) in terms of GDP generation, economic and social development is the strongest macroregion in Poland. In addition, due to the determinants of sustainable development, super macroregion II is metaphorically called the “Eastern Wall”. It refers to regions with unique natural resources—not only on a Polish, but also a European, and even a global scale.

The average HDI value of super macroregion II is relatively high and amounts to 0.884. This is due to the extremely high position of macroregion 7 on the national scale and valuable environmental resources of the other macroregions.

In addition to two super macroregions, cluster analysis also identified the third area, i.e., the southern macroregion (8.8% of Poland’s area). It consists of two regions (voivodships): Lesser Poland and Silesia. These are voivodships (two out of five) with the highest GDP in Poland in the long term.

Due to the high level of economic and social development of both regions, the southern macroregion has a higher average HDI value than both super macroregions i.e., HDI = 0.889.

Equally interesting is the fact that the HDI growth dynamics in the period 1995–2015 in the southern macroregion were the highest among the three newly delimitated spatial units, and amounted to 17.4%.

For the first super macroregion it was 12.5% and the second macroregion it was 12.6%.

Notably, the southern macroregion consists of only two regions with high parameters of socio-economic development.

The research objective adopted in the study was thus achieved. Three new spatial units consisting of macroregions (NUTS-1) were distinguished. They are characterized by a high degree of similarity in terms of sustainable development (the attributes of sustainable development were characterized by 27 variables divided into four pillars).

The accuracy of the obtained division results from the application of the kernel discriminant coordinates method. As a multidimensional method, it allows to achieve an extremely precise division of the examined objects.

**Figure 4:**
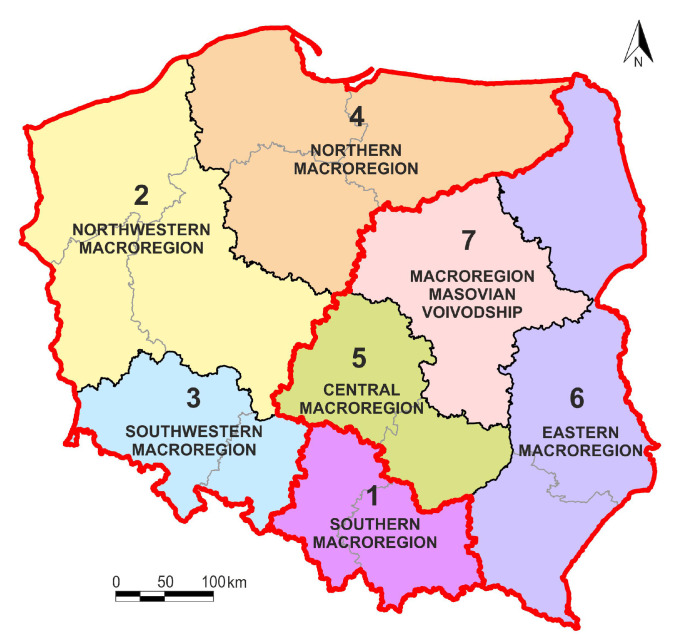
Super macroregions and macroregion of Poland. Source: own compilation.

Nevertheless, the super macroregions presented in Figure 4 should be treated as a scientific proposal subject to modification—taking into account other aspects of sustainable development and a different time interval.

## 5. Conclusions

This paper presented a proposal of structuring synthetic indicators for characterizing the sustainable development of macroregions described by spatio-temporal data. The values of kernel discriminant coordinates were adopted as these indicators. From a statistical point of view, this is an objective solution. The number of synthetic indicators is min(p,c−1), where *p* is the number of selected features characterizing the sustainable development of macroregions and *c* is the number of the studied macroregions. In the space of the first two indicators, one can see the mutual position of macroregions, while in the full space of the indicators, one can examine the strength differentiating macroregions by a selected set of features characterizing their sustainable development. The complete space of the indicators also allows to distinguish homogeneous subgroups of macroregions by means of the methods of cluster analysis. The operation of the proposed statistical methodology has been illustrated with spatio-temporal data concerning seven Polish macroregions.

The selection of 27 features characterizing the sustainable development of these regions turned out to be accurate, because in the six-dimensional space of synthetic indicators, errors in classifying 379 poviats to seven macroregions turned out to be zero or close to zero, depending on the selected statistical classifier. Consequently, the characteristics that optimally differentiate the studied macroregions were selected. The action of the proposed statistical methodology was illustrated with spatio-temporal data on seven Polish macroregions.

In the full six-dimensional space of synthetic indicators, three homogeneous subgroups of macroregions, called super regions, were separated using cluster analysis methods.

Although the data concern only one country, Poland, the presented statistical methodology is universal and can be applied to any territorial units in the world described by spatio-temporal data.

To the best of our knowledge, the proposed statistical methodology is new in the scientific literature. The justification may be the fact that we do not know any other solution for spatio-temporal data. In each study, the obtained results will always be a compromise between attempted assessment of the differentiation of the level of territorial units due to their sustainable development on the one hand, and the weakness of some diagnostic variables or the choice of the research method on the other hand.

Currently, research is being conducted on the construction of synthetic indicators describing the sustainable development of macroregions by replacing kernel discriminant coordinates with functional discriminant coordinates. Matrix data can be transformed into continuous functions (elements of a certain functional space). The transformed data are called functional data (Ramsay and Silverman [33], Górecki et al. [34]).

## Figures and Tables

**Table 1:** Structure of Polish macroregions including poviats.

Number	Name of the Macroregion	Number of Poviats in a Macroregion
1	Southern	58
2	Northwestern	70
3	Southwestern	41
4	Northern	64
5	Central	38
6	Eastern	66
7	Masovian voivodship	42

**Table 2:** List of the variables used in the research divided into pillars.

	Pillar		Variable
		1.	Population at working age
1.	Demography	2.	Femininity ratio
	and Social Capital	3.	Registered unemployment
		4.	Library books borrowers
		5.	General secondary schools
		6.	Primary schools
		7.	Pre-schools
		8.	Out-patient clinics
		9.	Pharmaceutical outlets
2.	Production,	10.	Libraries and branches
	Services and Trade	11.	Hotels, motels and boarding houses
		12.	Tourist accommodation establishments
		13.	The length of the water supply network
		14.	The length of the sewerage network
		15.	Boiler houses
		16.	Poviat hard surface roads
		17.	Poviats’ own revenue
3.	Public Finance	18.	General subsidies
		19.	Average monthly gross wages and salaries
		20.	Municipal biological wastewater treatment plants
		21.	Industrial biological wastewater treatment plants
		22.	Industrial chemical wastewater treatment plants
4.	Environment	23.	Industrial wastewater untreated, discharged
	and its Protection	24.	Forests
		25.	Renewals and afforestation—communal and private forests
		26.	Legal protected area
		27.	Monuments of nature

**Table 3:** Percentage of misclassification of poviats in the 6-dimensional space of KDC.

Number	Classifiers	10-cv (%)
1	LDF	0.00
2	NB (Normal)	0.79
3	KNN (K=2,…,8)	0.00
4	Tree (CART)	0.00
5	SVM	0.00

**Table 4:** Mahalanobis distances between macroregions.

Macroregion	1	2	3	4	5	6	7
1	0.0000	538.5115	631.1308	**717.4584**	588.5517	513.6408	**574.5919**
2	538.5115	0.0000	**128.1944**	**245.4427**	396.7969	339.3620	261.7809
3	631.1308	128.1944	0.0000	**295.5870**	340.7639	306.7737	199.0511
4	717.4584	245.4427	295.5870	0.0000	628.0889	579.4410	486.7190
5	588.5517	396.7969	340.7639	628.0889	0.0000	**81.6527**	**142.1345**
6	513.6408	339.3620	306.7737	579.4410	81.6527	0.0000	**117.2979**
7	574.5919	261.7809	199.0511	486.7190	142.1345	117.2979	0.0000

## References

[1] Awan U., Abbasi A.S., Humayon A.A. (2014). The Concept of Civic Sustainability is Need of Hour. Res. J. Environ. Earth Sci..

[2] Awan U., Krasławski A., Huiskonen J. (2018). Understanding influential factors on implementing social sustainability practices in Manufacturing Firms: An interpretive structural modelling (ISM) analysis. Procedia Manuf..

[3] Borys T. (1999). Indicators of Sustainable Development.

[4] Atkinson A. (1996). Developing indicators of sustainable community: Lessons from sustainable Seattle. Environ. Impact Assess. Rev..

[5] Bourdeau L. (1999). Sustainable development and future of construction: A comparison of visions from various countries. Build. Res. Inf..

[6] Spangenberg J.H. (2002). Environmental space and prism of sustainability: Frameworks for indicators measuring sustainable development. Ecol. Indic..

[7] (2008). OECD Territorial Reviews. Poland.

[8] Wheeler S.M., Beatley T. (2009). The Sustainable Urban Development.

[9] Wong C. (2006). Indicators for Urban and Regional Planning.

[10] Kontostanou-Karalivanou O., Maxon P.A., Sauerborn K., Scoullos M.J., Vonkeman G.H. (2000). Criteria and indicators for regional sustainable development. Sustainable Development of European Cities and Regions.

[11] United Nations (2007). Indicators of Sustainable Development: Guidelines and Methodologies.

[12] Streimikiene D. (2014). Comparative assessment indicators of quality of life in Romania and Lithuania. Econ. Sociol..

[13] Dočekalova M.P., Kocmanová A., Koleňák J. (2015). Determination of economic indicators in the context of corporate sustainability performance. Bus. Theory Pract..

[14] Jovovic R., Draskovic M., Delibasic M., Jovovic M. (2017). The concept of sustainable regional development- institutional aspects, policies and prospects. J. Int. Stud..

[15] Roszkowska E., Wyszkowska D. (2020). Spatial differentiation of the elderly population in Poland’s voivodeships in the years 2000–2016. Optim. Econ. Stud..

[16] Büyüközkan G., Karabulut Y. (2018). Sustainability performance evaluation: Literature review and future directions. J. Environ. Manag..

[17] Krasławski A., Huiskonen J., Awan U., Hussain C.M. (2019). Progress from blue to the green world: Multilevel governance for pollution prevention planning and sustainability. Handbook of Environmental Materials Management.

[18] Fisher R.A. (1936). The use of multiple measurements in taxonomic problems. Ann. Eugen..

[19] Rao C.R. (1948). The utilization of multiple measurements in problems of biological classification. J. R. Stat. Soc. Ser. B.

[20] Baudat G., Anouar F. (2000). Generalized discriminant analysis using a kernel approach. Neural Comput..

[21] Mika S., Ratsch G., Weston J., Schölkopf B., Müller K.R., Hu Y.H., Larsen J., Wilson E., Douglas S. (1999). Fisher discriminant analysis with kernels. Neural Networks for Signal Processing IX.

[22] Shawe-Taylor J., Cristianini N. (2004). Kernel Methods for Pattern Analysis.

[23] Krzyśko M., Waszak Ł., Wołyński W. (2014). Comparison of kernel discriminant coordinates algorithms. Commun. Stat. Simul. Comput..

[24] Cai D., He X., Han J. (2007). Efficient Kernel Discriminant Analysis via Spectral Regression.

[25] Zhang Z., Dai G., Xu C., Jordan M.I. (2010). Regularized discriminant analysis, ridge regression and beyond. J. Mach. Learn. Res..

[26] Seber G.A.F. (2004). Multiple Observations.

[27] Mirkin B. (2013). Clustering. A Data Recovery Approach.

[28] Krzyśko M., Wołyński W., Górecki T., Skorzybut M. (2008). Learning Systems: Pattern Recognition, Cluster Analysis and Dimensional Reduction.

[29] Wasserman L. (2004). All of Statistics. A Concise Course in Statistical Inference.

[30] Hastie T., Tibshirani R., Friedman J.R. (2009). The Elements of Statistical Learning: Data Mining, Inference, and Prediction.

[31] Kierczyńska A. Typology of Polish Poviats in View of the Variability of Economic and Social Development in 2002–2016.

[32] Hozer-Koćmiel M. (2018). Ocena rozwoju spoĺeczno-ekonomicznego województw za pomocą HDI (Assessement of the socio-economic development of voivodeships using HDI). WiadomoŚci Stat..

[33] Ramsay J.O., Silverman B.W. (2005). Functional Data Analysis.

[34] Górecki T., Krzyśko M., Waszak Ł. (2014). Functional discriminant coordinates. Commun. Stat. Theory Methods.

